# Polarized distribution of extracellular nucleotides promotes gravity-directed polarization of development in spores of *Ceratopteris richardii*

**DOI:** 10.3389/fpls.2023.1265458

**Published:** 2023-10-03

**Authors:** Ashley E. Cannon, Diana C. Vanegas, Tanya Sabharwal, Mari L. Salmi, Jeffrey Wang, Greg Clark, Eric S. McLamore, Stanley J. Roux

**Affiliations:** ^1^ Department of Molecular Biosciences, University of Texas at Austin, Austin, TX, United States; ^2^ Agricultural and Biological Engineering Department, The University of Florida, Gainesville, FL, United States

**Keywords:** extracellular nucleotides, Ceratopteris, gravity, polarization, gravity response, secretion, ion channels

## Abstract

Gravity directs the polarization of *Ceratopteris* fern spores. This process begins with the uptake of calcium through channels at the bottom of the spore, a step necessary for the gravity response. Data showing that extracellular ATP (eATP) regulates calcium channels led to the hypothesis that extracellular nucleotides could play a role in the gravity-directed polarization of *Ceratopteris* spores. In animal and plant cells ATP can be released from mechanosensitive channels. This report tests the hypothesis that the polarized release of ATP from spores could be activated by gravity, preferentially along the bottom of the spore, leading to an asymmetrical accumulation of eATP. In order to carry out this test, an ATP biosensor was used to measure the [eATP] at the bottom and top of germinating spores during gravity-directed polarization. The [eATP] along the bottom of the spore averaged 7-fold higher than the concentration at the top. All treatments that disrupted eATP signaling resulted in a statistically significant decrease in the gravity response. In order to investigate the source of ATP release, spores were treated with Brefeldin A (BFA) and gadolinium trichloride (GdCl_3_). These treatments resulted in a significant decrease in gravity-directed polarization. An ATP biosensor was also used to measure ATP release after treatment with both BFA and GdCl_3_. Both of these treatments caused a significant decrease in [ATP] measured around spores. These results support the hypothesis that ATP could be released from mechanosensitive channels and secretory vesicles during the gravity-directed polarization of Ceratopteris spores.

## 1 Introduction

Significant evidence supporting the hypothesis that extracellular nucleotides act as signaling molecules regulating plant growth and development has accumulated over the last decade. As reviewed in Clark and Roux (2018) and Pietrowska-Borek et al. (2020), extracellular nucleotides lead to an increase [Ca^2+^]_cyt_ and reactive oxygen species (ROS), and these secondary messengers lead to diverse downstream changes that affect plant defense, development, and growth.

The first eATP receptor to be identified in plants was discovered in *Arabidopsis*. In contrast to eATP receptors characterized in animal systems, the plant receptor, DORN1 (DOes not Respond to Nucleotides 1), is identical to a previously characterized lectin receptor kinase (LecRK-I.9/P2K1; At5g60300). It contains an extracellular lectin domain that, upon ATP binding, activates an intracellular kinase domain (Choi et al., 2014). In addition to P2K1, an additional eATP receptor was recently identified and named P2K2 (LecRK-I.5; At3g45430) (Pham et al., 2020). Although the P2K2 extracellular domain binds eATP with a higher affinity than P2K1, these two receptors, P2K1 and P2K2, interact and cross phosphorylate when eATP is applied to plants (Pham et al., 2020).

Studies of extracellular nucleotides and their role in plant growth and development have recently expanded to include a more primitive system, spores from the aquatic fern, *Ceratopteris richardii*. This single-cell, model system has been used to study gravity-directed polarization for decades. Its value as a model system is high because its development is well characterized (Chatterjee and Roux, 2000), it can be genetically transformed (Plackett et al., 2014; Bui et al., 2015; Plackett et al., 2015), and the genome has been sequenced (Marchant et al., 2022). Spore germination is initiated by water and red light. Within hours of initiation, calcium enters through channels at the bottom of spores and exits through pumps at the top (Ul Haque et al., 2007; Salmi et al., 2011). The calcium efflux peaks between 7 and 12 h after light-initiated germination begins (Chatterjee et al., 2000). Polarity of development is set by gravity after 24-30 h of growth and can be clearly visualized by the emergence of a downward-growing rhizoid 72 h after germination begins (Edwards and Roux, 1994).

The calcium differential described above is gravity-responsive and highly dynamic. When a spore is turned 180°, the calcium current re-orients within 24 seconds (Ul Haque et al., 2007; Salmi et al., 2011). The calcium differential is also sensitive to the magnitude of the *g*- force (Ul Haque et al., 2007; Salmi et al., 2011). During parabolic flight, when spores were exposed to 2*g*, the magnitude of the calcium differential increased. When spores are exposed to micro-*g* on the same flight, the calcium differential decreased to baseline levels. Additionally, blocking calcium channels with nifedipine decreased the number of downward growing rhizoids (Chatterjee et al., 2000). All of these data point to the significance of the calcium current and its role in gravity-directed polarization.

To further understand the molecular mechanisms associated with gravity- directed polarization, Bushart et al. (2013) used an RNA-SEQ analysis to identify components of the transcriptome that influence or respond to the calcium current. In this study, a key sequence found was one that, when translated, had apyrase conserved domains and a 59% identity with an *Arabidopsis* apyrase that is postulated to help control the concentration of extracellular nucleotides (Lim et al., 2014). Additionally, a western blot using polyclonal antibodies raised to *Arabidopsis* apyrase confirmed the presence of an immunoreactive protein band of the predicted apyrase size in extracts of *Ceratopteris* spores (Bushart et al., 2013). Because apyrase expression can be induced by an increase in eATP (Wu et al., 2007), this discovery suggested the possibility that extracellular nucleotides could be playing a role in the gravity-directed polarization of spore cells.

As an initial test of this hypothesis, an assay of the ATP released by spores into the growth media was done, and it showed that extracellular ATP (eATP) accumulates during gravity-directed polarization of *Ceratopteris* spores (Bushart et al., 2013). Because eATP can promote the uptake of calcium into cells (Jeter et al., 2004; Demidchik et al., 2009; Demidchik et al., 2011), these results raised the question of whether the release of ATP from spores was polarized, and, if so, whether this eATP could contribute to the gravity-induced polarized entry of calcium along the bottom of the spores. These questions are addressed in this study, using an amperometric ATP biosensor (Vanegas et al., 2015) to measure the [eATP]. The novel results presented here are consistent with the hypothesis that gravity induces the polarized release of ATP from cells, and that the consequent asymmetric accumulation of eATP could potentially help induce one of the earliest steps in gravity signaling, the polarized entry of calcium into cells, which polarizes cell development.

## 2 Materials and methods

### 2.1 Plant material and growth conditions

*Ceratopteris richardii* spores were surface sterilized by soaking in 20% bleach for 1.5 min and rinsed three times with sterile water. After sterilization, spores were soaked in sterile water in the dark at 28°C for 5-7 days in order to increase synchronization of development (**Supplementary Figure S1**). Spores were induced to germinate in an incubator set at 28°C with illumination from two 100W equivalent daylight (5000K) LED bulbs located on one side of the chamber. Light was given continuously throughout the germination period. The fluence rate of the incident light was ca. 180 µmol/m^2^s.

### 2.2 ATP flux measurements in *Ceratopteris* spores

The protocol outlined in Vanegas et al. (2015) was used for fabricating biosensors and directly measuring surface ATP concentration during gravity-directed polarization in *Ceratopteris* spores. Specifically, after soaking for 7 days in the dark, spores were immediately used for measurements or grown at 28°C for 16 – 22 h in an incubator and then used for ATP measurements. Prior to measuring ATP, spores were immobilized on 100 μm mesh and submerged in liquid ½-strength MS media (Caisson Labs), pH 6.3 with 0.1% glycerol. The spores were maintained in a vertical orientation on the mesh for 30 min prior to measurements in order to ensure that the ATP measured was not due to mechanostimulation. The measurements of ATP concentration were taken at the surface of germinating spores (n ≥ 6) for 10 min at both the bottom and top of each spore. A stereo zoom scope and computer-controlled stepper motors were used to position the micro-biosensor approximately 1-2 μm from the spore surface. ATP calibration tests were made in the presence of GdCl_3_ (1 mM) and BFA (5 µM) to determine whether these drugs alter the electrochemical performance of the micro-biosensor, thus allowing for accurate determination of [eATP] in the presence and absence of the chemicals used. The top and bottom of spores were measured prior to the chemical treatment (sensors were calibrated before and after each experiment). The chemical was applied, and measurements of the same spores were taken 1 h later (**Supplementary** **Figure S1**).

### 2.3 Pharmacological treatment of *Ceratopteris* spores

After they were soaked in darkness, spores were exposed to light and rinsed three times with sterile water. Then ½-strength MS medium (Sigma-Aldrich), pH 6.3 with 1.25% Noble agar (Difco) was added in order to achieve a final spore density of 1 mg spores/mL of medium. Spores were sown on microscope slides that had been lightly sanded on one side in order to help the spores and agar better adhere to the slide and prevent them from slipping off. After the agar solidified for approximately 5–10 min, the slides were immersed in 20 mL of liquid ½ MS medium, pH 6.3 in a slide box. Pharmacological treatments (Sigma-Aldrich) were added to the liquid media prior to pouring it into each slide box. The slides were maintained in each treatment for 30 h. After 30 h, the spores were rinsed with liquid ½-strength MS, twice, with at least 1 hour between each rinse. Slides remained in this medium until visualization and assessment of polarized rhizoid emergence at approximately 90 h. After each experiment was set up, the slide boxes were placed in an incubator set at 28°C with constant light until polarization and germination assessment (**Supplementary F** **igure S1**).

### 2.4 Polarization and germination assessment

After spores had grown for at least 90 h, germination and polarization due to gravity was assessed (**Supplementary F** **igure S1**). Spores were considered germinated if at least one rhizoid that was at least one spore diameter long was present, or if a prothallus was present. In order to quantitatively assess gravity-directed polarization, the percentage of downward-growing primary rhizoids was determined. If the rhizoid was growing below a virtual horizontal line drawn through the center of the spore, the rhizoid was considered as growing downward, and if the rhizoid was growing above this line, it was considered as growing upward. Rhizoids growing along this line were considered as growing sideways, and not growing downward.

## 3 Results

### 3.1 Gradient of eATP during polarization

**Figure 1:**
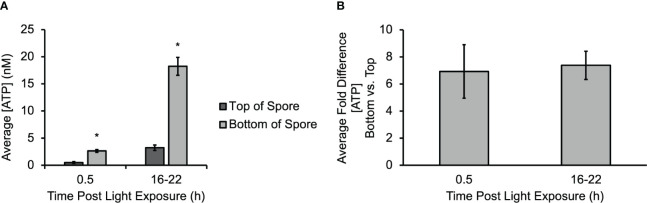
A gradient of eATP develops and persists throughout polarization. **(A)** The gradient of eATP is evident within the first 30 min of development and continues for at least the first 22 h The [ATP] released at the top and bottom of the spore is significantly higher after 16-22 h The asterisk indicates statistical significance as determined by a one-way ANOVA (F (3,34) =2.88, p= 1.3x10^-10^) and *post-hoc* tests using the Tukey-Kramer test. **(B)** The average fold difference between the bottom and the top after 0.5 h and 16-22 h The fold differences are not statistically different between 0.5 h and 16-22 h (Student’s t-test, p > 0.05). Error bars represent standard error of the mean. (n=3 for 0.5 h and n=16 for 16-22 h). Asterisks represent significant differences and error bars show standard error of the mean.

A micro-biosensor (Vanegas et al., 2015) was used to directly measure ATP release at different regions of *Ceratopteris* spores during gravity-directed polarization. The average sensitivity of the biosensor before experiments (4.03 ± 0.82 pA nM^-1^) was not statistically different than after experiments (3.79 ± 0.33 pA nM^-1^). An additional control study for the biosensor calibration was performed in the presence of an amino acid (glutamate) and two organic acids (ascorbic acid and citric acid) to ensure no false positives contributed to the data; see **Supplementary Figures S2, S3** for calibration data. **Figure 1A** shows the average concentration of eATP measured on both the top and bottom of multiple spores after 30 min and 16-22 h of light exposure. The [eATP] measured along the bottom of the spore was statistically significantly higher as early as 30 min after light exposure and throughout gravity-directed polarization. After 30 min of light exposure, the [eATP] measured along the bottom of the spore ranged from 2.2 to 3.2 nM. The [eATP] measured along the top of the spore during the same period ranged from 0.3 to 0.9 nM. After 16 to 22 h of germination, the [eATP] measured at the bottom of the spore ranged from 10.3 to 35.4 nM, and the [eATP] measured at the top of the spore ranged from 1.0 to 7.6 nM after the same amount of time. Approximately 10% of the spores had [eATP] levels that were not detectable on the top (the detection limit of the biosensor was 1.0 ± 0.8 nM), and these data were not included in the average values reported above. **Figure 1B** shows that the average fold difference when the [eATP] along the bottom is compared to the concentration along the top is 6.9 at the beginning of germination. Over time, as the concentration of ATP released from spores increased, the fold difference went up to an average of 7.4-fold after 16-22 of light exposure (**Figure 1B**). Both at 0.5 h and at 16-22 h the fold differences between the top and bottom were significant, but the 6.9-fold and 7.4-fold differences at these two time points were not significantly different. Overall, these data show that ATP is released during gravity directed polarization both from the bottom and top of germinating *Ceratopteris* spores and that the quantity of ATP released increases significantly over time. The data also show that the [ATP] released from the bottom of a spore is significantly higher than the [ATP] released from the top (**Figure 1A**).

### 3.2 Pharmacological disruption of eATP gradient decreases gravity-directed polarization

**Figure 2:**
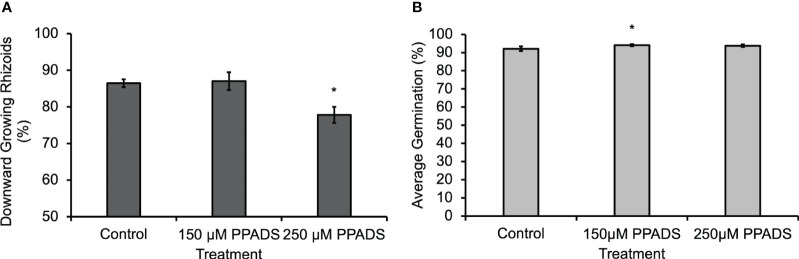
*Ceratopteris* spores treated with 250 μM PPADS showed a significantly lower percentage of downward growing rhizoids. **(A)** Treating *Ceratopteris* spores with 250 μM PPADS caused a significant decrease in the percentage of downward growing rhizoids. An asterisk indicates a significant difference as determined by the one-way ANOVA (F (2,15) = 6.96, p=0.007) and *post-hoc* tests using the Tukey-Kramer test. **(B)** Spores treated with 150 μM PPADS had a significantly higher percentage of germination. There was a significant difference in the germination determined by a one-way ANOVA (F (2,15) = 5.69, p=0.01) and *post-hoc* tests using the Tukey-Kramer test. Asterisks represent significant differences and error bars show standard error of the mean.

In order to determine if the eATP gradient is physiologically relevant to gravity-directed polarization, multiple chemical reagents were used to either block eATP effects or disrupt the gradient. When a purinoceptor antagonist, Pyridoxal phosphate-6-azo (benzene-2,4-disulfonic acid) (PPADS), was applied to spores for 24 h, or when the nucleotide gradient was disrupted by flooding the medium with 150 µM ATPγS, a poorly hydrolysable form of ATP, for 24 h, there was a statistically significant decrease in the gravity response of *Ceratopteris* spores (Bushart et al., 2013). Following up on this result, *Ceratopteris* spores were treated with lower concentrations of PPADS in order to determine the effective threshold concentration. The results showed that the PPADS concentration required to disrupt the gravity response was between 150 μM and 250 μM (**Figure 2A**). Neither 150 μM nor 250 μM PPADS decreased spore germination (**Figure 2B**). These treatments support the conclusion that eATP signaling, possibly mediated by an unknown receptor, is important for the gravity response in Ceratopteris spores.

**Figure 3:**
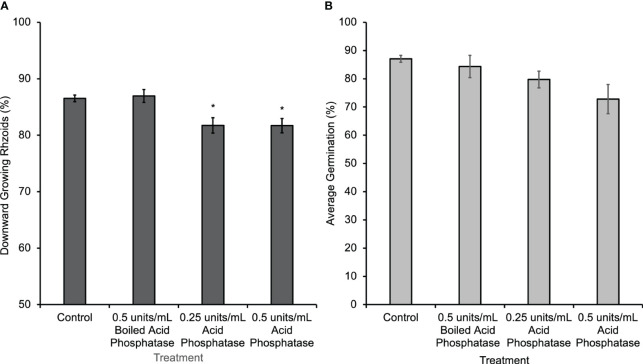
*Ceratopteris* spores treated with acid phosphatase from wheat germ for 30 h had a significant decrease in the percentage of downward growing rhizoids. **(A)** The average percentage of downward growing rhizoids decreased when spores were treated with acid phosphatase. The asterisks indicate that there was a significant difference between groups as determined by a one-way ANOVA (F (3,69) =7.52, p=0.0002) and *post-hoc* tests using the Tukey-Kramer test. When acid phosphatase is boiled, it has no effect on polarization. **(B)** There was no statistical difference in percent germination between groups as determined by the one-way ANOVA (F (3,28) =2.95, p=0.07). Asterisks represent significant differences and error bars show standard error of the mean.

A second method of disrupting the gradient was to eliminate it enzymatically by hydrolyzing the eATP with phosphatase. Wheat germ acid phosphatase was applied only during the first 30 h of gravity-directed polarization. This treatment caused a subtle but significant decrease in gravity-directed polarization in *Ceratopteris* spores (**Figure 3A**). Acid phosphatase treatment significantly decreased the percentage of downward growing rhizoids, but when this enzyme was boiled and applied to spores there was no change in gravity-directed polarization or germination (**Figures 3A, B**).

### 3.3 ATP could be released from mechanosensitive channels or secretory vesicles

**Figure 4:**
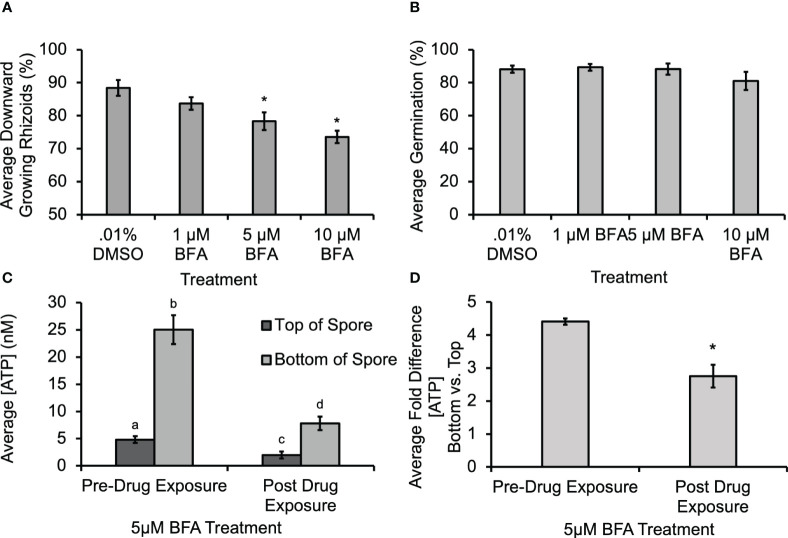
Treatment with 5 μM and 10 μM Brefeldin A (BFA) caused a significant decrease in the gravity response and in the [ATP] measured near *Ceratopteris* spores. **(A)** A 30-h treatment with 5 μM and 10 μM BFA caused a significant decrease in the percentage of downward growing rhizoids. An asterisk shows that there was a significant difference in the gravity response when spores were treated with BFA as determined by a one-way ANOVA (F (3,34) =2.88, p=2.6x10^-5^) and *post-hoc* tests using the Tukey-Kramer test. **(B)** The was no statistical difference in percent germination as determined by the one-way ANOVA (F (3,34) = 2.88, p=0.12). **(C)** Treatment with 5μM BFA caused a significant decrease in [ATP] measured near *Ceratoperis* spores during gravity-directed polarization. A one-way ANOVA (F (3,20) =3.10, p= 2.0x10^-16^) and *post-hoc* tests using the Tukey-Kramer test showed that there was a significantly higher [ATP] around the bottom of spores compared to the top both pre- and post-drug exposure and that the [ATP] released after 1h of 5 μM BFA treatment was significantly lower. **(D)** The average fold difference between the bottom and the top after BFA treatment. The fold differences are statistically different between pre- and post-drug exposure (Student’s t-test, p < 0.00). Error bars represent standard error of the mean. Letters and asterisks represent significant differences and error bars show standard error of the mean.

Previous studies documented that ATP can be released from angiosperm cells through secretory activity (Kim et al., 2006; Wu et al., 2007), or through mechanical stimulation of cell membranes (Jeter et al., 2004; Weerasinghe et al., 2009). To test whether secretory activity helped to mediate ATP release from fern cells, spores were treated with Brefeldin A (BFA), which could disrupt secretion, including the delivery of Ca^2+^-permeable channels (such as, e.g., MS channels), to the membrane. The BFA caused a significant decrease in gravity-directed polarization (**Figure 4A**), but it did not cause a statistically significant decrease in germination when 1 µM, 5 µM or 10 µM was applied during the first 30 h of development (**Figure 4B**). Control studies were conducted to test whether the biosensor was responsive to BFA; there was a shift in baseline oxidative signal, but no change in the calibration slope. Thus, all [eATP] measurements were corrected for this as shown in **Supplementary Figure S4**. The [ATP] released from *Ceratopteris* spores during gravity-directed polarization decreased when spores were grown in the presence of 5 μM BFA (**Figure 4C**). This treatment also significantly decreased the average fold difference from 4.6 to 2.8 (**Figure 4D**).This result is consistent with the hypothesis that BFA treatment causes a disruption in gravity-directed polarization due to a decrease in the level of ATP released from spores. However, the decrease in [eATP] may not be a direct result of inhibiting the secretion of this signaling molecule, but instead could be caused by the inhibition of secretion of another component involved in eATP signaling.

**Figure 5:**
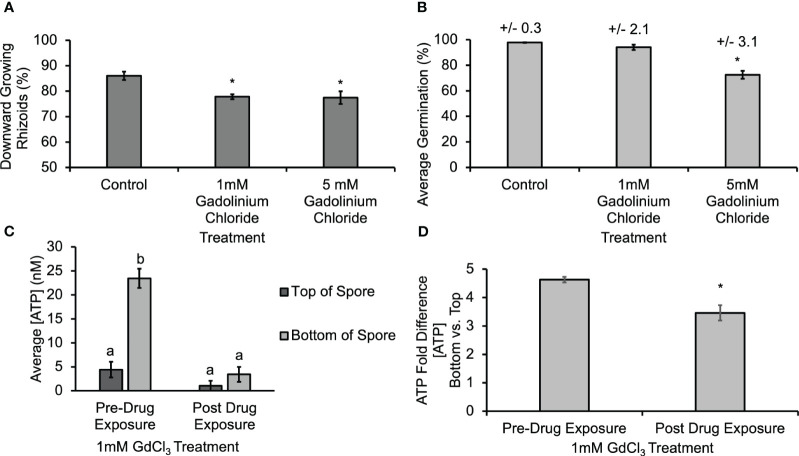
Treatment with 1mM and 5mM GdCl_3_ caused a significant decrease in the gravity response of *Ceratopteris* spores, and 1mM GdCl_3_ also decreased the [ATP] measured near spores. **(A)** A 30 h treatment with 1 mM and 5 mM GdCl_3_ caused a statistically significant decrease in the percentage of downward growing rhizoids. Statistical significance was determined by a one-way ANOVA (F (2,28) =3.34, p=0.0001) and *post-hoc* tests using the Tukey-Kramer test. Asterisks represent a statistical difference between groups. **(B)** Treatment with 5 mM GdCl_3_ caused a significant decrease in germination as determined by the one-way ANOVA (F (2,22) =3.44, p= 2.1x10^-5^) and *post-hoc* tests using the Tukey-Kramer. **(C)** Treatment with 1mM GdCl_3_ caused a significant decrease in the [ATP] near *Ceratopteris* spores during gravity directed polarization. A one-way ANOVA (F (3,20) =3.10, p= 2.0x10^-16^) showed that there was a difference in the [ATP] near *Ceratopteris* spores and *post-hoc* tests using the Tukey-Kramer test revealed that there was a significantly higher [ATP] near the bottom of spores compared to the top pre-drug exposure and that after 1 h of 1 mM GdCl_3_ treatment [ATP] was significantly lower and the gradient was no longer present. **(D)** The average fold difference between the bottom and the top after 1mM GdCl_3_treatment. The fold differences are statistically different between pre- and post-drug exposure (Student’s t-test, p < 0.00). Error bars represent standard error of the mean. Letters and asterisks represent statistically significant differences and error bars show standard error of the mean.

Gravitational stimuli are known to induce the opening of mechanosensitive channels (Nakano et al., 2021), and gadolinium ions are known to block mechanosensitive channels (Ermakov et al., 2010) and calcium channels (Lewis and Spalding, 1998). In order to determine whether the activity of MS channels and calcium channels might help mediate the gravity-directed polarization, spores were also treated with 1 mM and 5 mM GdCl_3_ during the first 30 h of development. This treatment resulted in a statistically significant decrease in the gravity response of spores (**Figure 5A**). Additionally, treatment with 5 mM GdCl_3_ caused a statistically significant decrease in germination (**Figure 5B**). Similarly, to the results in **Supplementary Figure S4**, biosensor calibrations were conducted in the presence of GdCl_3_, and [eATP] data was corrected for the baseline artifact caused by GdCl_3_. The [ATP] released from *Ceratopteris* spores during gravity-directed polarization decreased and the gradient between the top and the bottom was abolished when spores were grown in the presence of 1 mM GdCl_3_ (**Figure 5C**). These data are consistent with the hypothesis that ATP released from MS channels promotes gravity-directed polarization in *Ceratopteris* spores. These data also support a role for calcium channels in this developmental process. The role and significance of a gravity-directed calcium current in the polarization of Ceratopteris fern spores has been published previously (Chatterjee et al., 2000). However, this is the first report of a gadolinium-sensitive eATP gradient and together these results support the hypothesis that a eATP gradient and calcium differential promote gravity-directed polarization in Ceratopteris spores. Conversely, the degradation of ATP caused by an indirect result of inhibiting MS channels may also be the cause of a decrease in [eATP].

**Figure 6:**
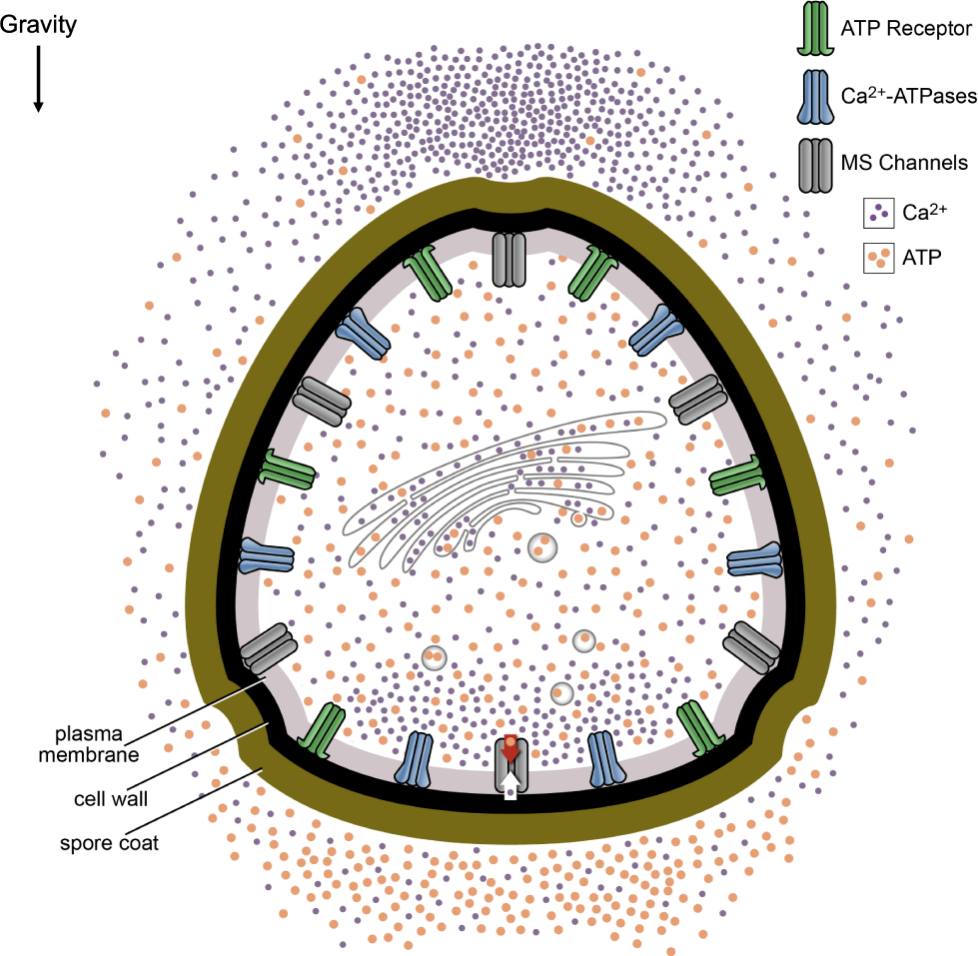
Model of eATP and calcium signaling in early growth and development of *Ceratopteris* spores. The model predicts that ATP is released from MS ion channels as a result of changes in membrane tension and secretory vesicles delivering growth materials to the plasma membrane. In the model, the settling of the protoplasm or unidentified cellular bodies in response to gravity causes the opening of MS ion channels. Because these channels are preferentially opened along the bottom of the spore, the ATP release would create a gradient where the highest [eATP] would be predicted to be at the bottom of the spore. This eATP could be contributing to the Ca^2+^ differential by opening additional Ca^2+^ channels. In addition to releasing ATP, the MS ion channels may be the initiators of Ca^2+^ uptake in *Ceratopteris* spores during gravity directed polarization. Ca^2+^ is taken up by MS channels selectively activated at the bottom of the spore which may lead to an intracellular accumulation of Ca^2+^ near the bottom of the spore.

In the model, shown in **Figure 6**, ATP is released from MS ion channels activated along the bottom of the spore in response to gravity and from secretory vesicles delivering growth materials to the plasma membrane. This asymmetric release of ATP leads to an accumulation along the bottom of the spore. When the ATP at the bottom of the spore surpasses a threshold, this would allow ATP receptors to be activated. The activated ATP receptors directly or indirectly open additional calcium channels. By opening additional calcium channels, the eATP gradient is promoting gravity-directed polarization by increasing the magnitude of the calcium differential.

### 3.4 Identification of a putative Msc channel in *Ceratopteris* spores

Partial transcripts encoding a putative MS channel were identified in the two transcriptome sequences of *Ceratopteris* spores reported in Salmi et al. (2005) and Bushart et al. (2013). A full-length transcript, which included the partial region previously identified, was kindly provided by Andrew Plackett. Based on this sequence, primers to the full-length transcript were designed and used to amplify and clone the transcript. The complete coding region from sequencing this cloned product is available through GenBank. This transcript encodes a 516 amino acid protein, which includes the canonical MS channel domain shared by all proteins in the family of mechanosensitive channels of small (MscS) conductance (**Supplementary Table S1**). Phytozome was used to identify longer protein sequences of the three MS channels shown in **Supplementary** **Table S1** (Goodstein et al., 2012; Marchant et al., 2022). These sequences were then used to identify similar protein sequences in *Arabidopsis thaliana*, *Chlamydomonas reinhardtii*, and *Oryza sativa.* Multi-sequence alignments between these protein sequences are included in **Supplementary Figures S5–S7** (Brown et al., 1998; Larkin et al., 2007). In these alignments, Ceratopteris sequences have the highest percent identity when compared to *Arabidopsis thaliana* sequences. The BLAST search using Ceratopteris MscC and MscD picked up mitochondrial and chloroplast homologs, respectively. In addition, analysis of Ceratopteris Msc candidate transcript abundance after 6 hours and 24 hours of light exposure was also analyzed and included in **Supplementary F** **igure S8**. Collectively, these data support the 454 data and provide additional preliminary data that support the presence of MS channels in Ceratopteris that are expressed during polarization of development.

## 4 Discussion

To determine if eATP is released from germinating spores during gravity-directed polarization, an ATP-selective micro-biosensor was used to directly measure ATP release from germinating spores. Calibrations were conducted before/after each experiment, and in the presence of amino acids, organic acids, BFA, and GdCl_3_ to ensure no calibration drift caused bias in the data (see **Supplemental Section** for data). This assay showed that ATP was released from spores during their gravity-directed polarization, and that this release occurs as early as 30 min after light exposure, nearly coincident with the earliest detection of a gravity-induced calcium differential. The data presented here favor the hypothesis that the asymmetric accumulation of eATP preferentially along the bottom of germinating spores helps promote the gravity-directed polarization of these cells. Because eATP promotes the uptake of calcium in both animal and plant cells, we postulate that one potential role of eATP in spore cell polarization is to enhance the entry of calcium into the spores, and thus promote the trans-cell calcium current that orients cell polarization. Although eATP does induce calcium entry into Arabidopsis cells (Jeter et al., 2004; Demidchik et al., 2009), whether it can do so also in Ceratopteris cells remains to be tested.

In our previous work, we showed that the accumulation of ATP outside of spore cells was temporally dynamic (Vanegas et al., 2015), meaning that although there was a net efflux of ATP, this signaling molecule cyclically peaks in concentration every 10-12 min followed by a decrease in its concentration at the surface of the spores for 6-9 min. The temporally dynamic nature of ATP accumulation is evidence of modulation of the [eATP], presumably by ecto-phosphatases. The best characterized of these enzymes are ecto-apyrases, the phosphatases with the lowest Km for ATP thus far reported (Knowles, 2011). Maintaining precise spatial and temporal control of eATP during development ensures that a pivotal cellular decision dependent on the presence of this signal, such as polarization, is tightly regulated. Although the [eATP] dynamically changes, there is also a net increase in the accumulation of this molecule over time. This increase in accumulation may point to the necessity to surpass a threshold level in order to activate calcium channels. The opening of additional calcium channels would lead to an increase in the uptake of calcium and an increase in the accumulation of intracellular calcium along the bottom of the spore, promoting polarization.

Since a gradient of eATP is present as early as 30 min after light exposure and persists throughout polarization, the physiological relevance of this gradient was explored. That eATP could play a role in the spore gravity response was first shown in Bushart et al. (2013), who found that adding to the growth medium either a high concentration of extracellular nucleotides or an extracellular ATP receptor antagonist caused a statistically significant decrease in gravity directed polarization. Based on these results, Bushart et al. (2013) postulated that if gravity induced an asymmetric release of ATP this could help polarize the spore cells. The data presented here provide support for that postulate.

Blocking eATP responses with 350 μM PPADS significantly reduced the effect of gravity on spore polarization (Bushart et al., 2013). In the experiments shown in **Figure 2A**, both 150 μM and 250 μM PPADS were applied to spores in order to determine if a lower concentration would also disrupt the gravity response in *Ceratopteris* spores. Based on the results shown in **Figure 2A**, the eATP receptor antagonist threshold is 250 μM or between the two concentrations tested because 150 μM had no effect on polarization.

Hydrolyzing the eATP gradient using acid phosphatase (**Figure 3A**) or apyrase caused a small but statistically significant decrease in the gravity response in this system. This result suggests two alternative hypotheses that could explain the subtleness of the physiological response to this treatment. First, given the relatively large size of acid phosphatase (58 kD) and the denseness of the thick spore coat, only very low levels of these enzymes would be likely to penetrate through to the plasma membrane. This would limit the access of the enzyme to the site of eATP action and thus limit its effect on the eATP gradient and on gravity-directed polarization. In addition, since the Km of acid phosphatase is in the µM range, this treatment may not have a substantial effect because the concentration of ATP released into the external medium by spores is in the nM range. The alternative hypothesis is that eATP is promoting gravity-directed polarization rather than initiating it. According to this second hypothesis, in the absence of an extracellular nucleotide gradient, most spores would still polarize in response to gravity. However, if more eATP accumulated at the bottom pole of the cell, additional calcium channels would be opened there, causing an increase in the uptake of calcium and an increase in the percent of spores polarizing in response to the increased calcium channel activity.

In order to begin investigating the source of ATP release during polarity fixation, spores were treated with BFA and GdCl_3_. In **Figures 4A**, **5** μM BFA treatment caused a statistically significant decrease in gravity-directed polarization. Since ATP can be released by both MS channels and as a result of secretory vesicles fusing with the membrane, this treatment does not differentiate between the two mechanisms, but the result in **Figure 4A** could be showing the necessity of ATP release by one or both of them. The BFA also caused a statistically significant decrease in the [eATP] measured on the top and bottom of *Ceratopteris* spores during gravity-directed polarization (**Figure 4C**). Additionally, the fold-change between the bottom and the top of spores decreases significantly, perhaps enough to fall below a hypothetical threshold. A decrease in ATP release as a result of BFA treatment has previously been reported in growing root hairs of *Arabidopsis thaliana* (Kim et al., 2006).

The release of ATP could also be mediated by MS channels during gravity-directed polarization in *Ceratopteris* spores. Treating spores with a MS channel antagonist, GdCl_3_, caused a statistically significant decrease in the gravity response (**Figure 5A**). In addition, treating spores with 1 mM GdCl_3_ decreased the [eATP] measured on the top and bottom of spores during gravity-directed polarization. Unlike BFA, GdCl_3_ treatment disrupted the ATP gradient (**Figure 5C**). Treatment of *Ceratopteris* spores with GdCl_3_ will also cause a decrease in the uptake of Ca^2+^ through MS channels, and, as noted by Kim et al. (2006), a decrease in Ca^2+^ uptake would decrease the [ATP] released. These results identify two potential mechanisms of ATP release during the gravity response in *Ceratopteris* spores, secretion and MS channels.

Overall, the significant decrease in gravity-directed polarization after BFA and GdCl_3_ treatment, the lower [ATP] released from spores as a result of BFA and GdCl_3_ treatments, and the significant decrease in the average fold difference as a result of BFA and GdCl_3_ treatments are consistent with a hypothetical model that predicts the release of ATP by MS channels and targeted secretion will promote gravity-directed polarization in *Ceratopteris* spores (**Figure 6**). The transcript encoding a putative MS channel that has been identified in the transcriptome of *Ceratopteris* spores (**Supplementary T** **able S1**) is to date the only one confirmed to be expressed during the polarization period of development. Stronger support for the predictions of **Figure 6** would require identifying the MS channel(s) that are localized on the plasma membrane in *Ceratopteris* and then showing that the knockout of this channel (or these channels) blocks graviresponsiveness of the spore.

Multiple experiments using *Ceratopteris* spores support the idea that an electrical gradient is generated by the asymmetrical distribution of Ca^2+^ during gravity-directed polarization (Chatterjee et al., 2000; Ul Haque et al., 2007; Salmi et al., 2011). This electrical gradient is essential for gravity-directed polarization in these spores (Chatterjee and Roux, 2000). The hypothetical model generated and supported by the experiments outlined in this paper support the hypothesis that ATP released from MS channels, secretory vesicles, or both is promoting the uptake of Ca^2+^ necessary to generate an intracellular electrical gradient. This hypothetical intracellular electrical gradient could be the initiating signal that sets up that polarization of early growth and developmental steps in *Ceratopteris* spores, including nuclear migration, asymmetric cell division, and emergence and growth of a rhizoid in the downward direction in response to gravity. Previous studies in zygotes of *Fucus* and *Pelvetia*, brown algae, have shown that the formation of an intracellular Ca^2+^ gradient is essential for directed polarization (Robinson, 1996). These studies support the hypothesis that the extracellular Ca^2+^ gradient previously measured in *Ceratopteris* spores (Chatterjee et al., 2000; Ul Haque et al., 2007; Salmi et al., 2011) is suggestive of an intracellular gradient that is necessary for gravity-directed polarization.

The idea of a differential distribution of a signaling molecule leading to morphological polarity is not unique. In this regard, gradients of auxin are among the best documented to be inductive of cell polarity (Dhonukshe, 2009). The differential distribution of eATP during gravity-directed polarization could be an early step in a series of asymmetrical molecular mechanisms that ultimately lead to the downstream cellular and morphological changes needed to generate a gravity-directed, downward growing rhizoid in Ceratopteris spores.

Precedent data relevant to this hypothesis are those of Tang et al. (2003), who showed that a high [eATP] inhibits the gravity response in Arabidopsis roots. They interpreted their data as evidence that an eATP gradient could be one of the first steps involved in the gravity response in plant cells, and, in support of that interpretation, they showed that high levels of eATP decreased shoot-ward auxin transport in a dose-dependent manner, leading to an inhibition in the gravity-response.

Like applied ATP, applied brefeldin and gadolinium also block gravity responses in roots (Abas et al., 2006; Soga, 2013), and all three of these agents would also be expected to affect, directly or indirectly, tip-focused calcium gradients that are characteristic of polarly growing cells and required for polarized growth (Campanoni and Blatt, 2007; Rincon-Zachary et al., 2010). Taken together these data underscore the likelihood that signaling components of the gravity response have been conserved in evolution from ferns through flowering plants.

These results show a clear role for eATP in gravity-directed polarization of Ceratopteris spores. However, there are still questions remaining about the molecular components and downstream mechanisms involved in this response. Although data support the idea that eATP could open Ca2+ channels leading to a higher influx during polarization, additional experiments are required to show a clear link between eATP and Ca2+ influx. Nonetheless, our data reveal that quantifying and evaluating gradients of eATP can lead to a more thorough understanding of how plants use ATP release to direct growth and development.

## Data availability statement

The datasets presented in this study can be found in online repositories. The names of the repository/repositories and accession number(s) can be found below: https://www.ncbi.nlm.nih.gov/genbank/, KX961683.

## Author contributions

AC: Conceptualization, Investigation, Methodology, Supervision, Validation, Writing – original draft, Writing – review & editing. DV: Formal Analysis, Investigation, Methodology, Validation, Writing – review & editing. TS: Investigation, Writing – review & editing. MS: Funding acquisition, Investigation, Methodology, Supervision, Writing – original draft, Writing – review & editing. JW: Formal Analysis, Investigation, Writing – review & editing. GC: Conceptualization, Formal Analysis, Funding acquisition, Investigation, Methodology, Resources, Supervision, Writing – original draft, Writing – review & editing. EM: Conceptualization, Formal Analysis, Investigation, Project administration, Resources, Supervision, Writing – original draft, Writing – review & editing. SR: Conceptualization, Funding acquisition, Methodology, Project administration, Supervision, Writing – original draft, Writing – review & editing.
