# Supplementary material for: Polarized distribution of extracellular nucleotides promotes gravity-directed polarization of development in spores of Ceratopteris richardii
Source: Front Plant Sci. 2023 Oct 3;14:1265458. doi: 10.3389/fpls.2023.1265458 (PMC10579945; doi:10.3389/fpls.2023.1265458)
Supplement: Supplementary file 1 [file DataSheet_1.docx]

**Supplemental Figures**

**
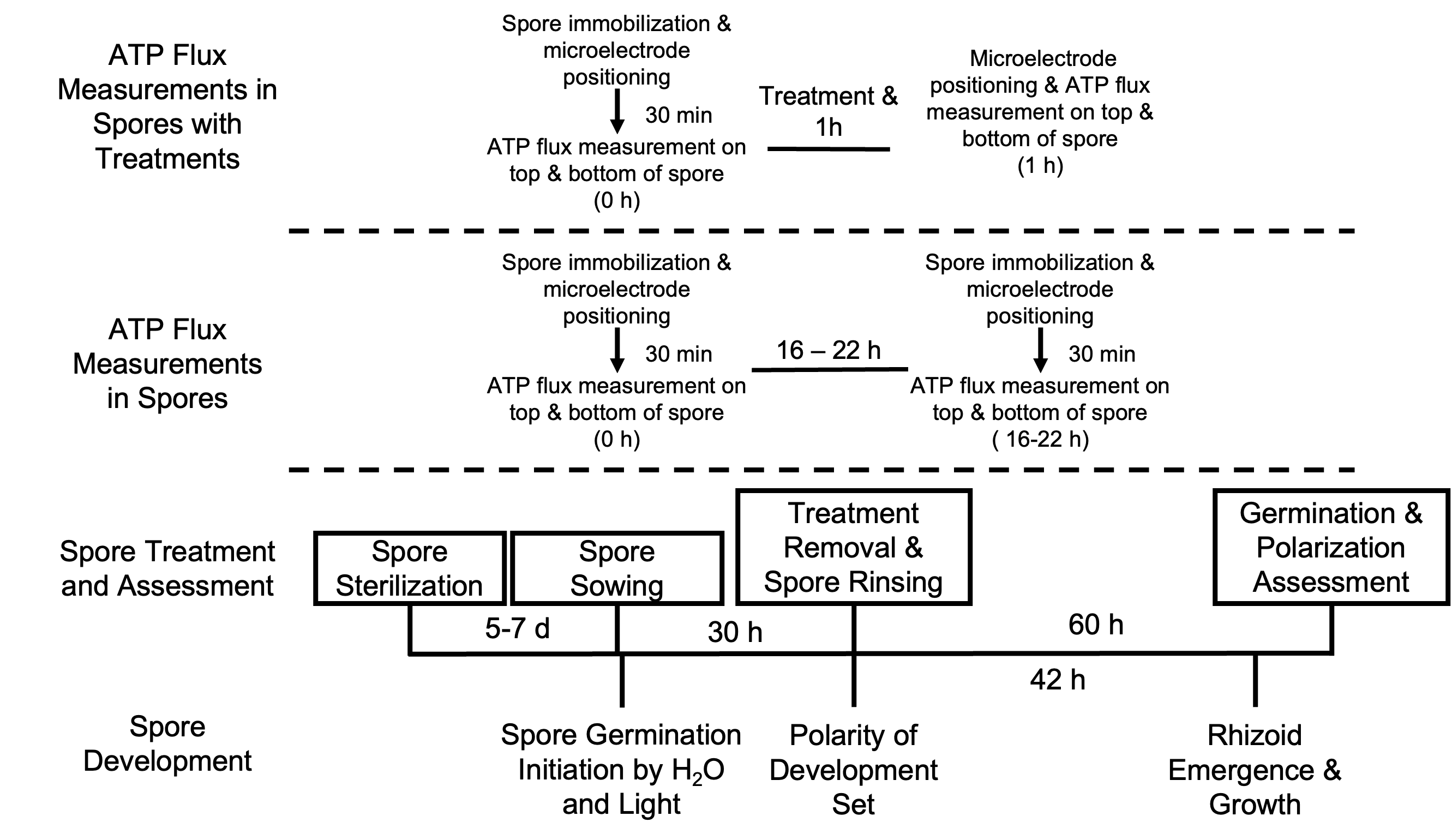
**

**Figure S1**. Experimental timeline(s) and developmental events in Ceratopteris spores. ATP flux measurements were taken during the developmental period when spores are using environmental stimuli to determine the polarity of future developmental events. In addition, treatments were applied during this period. After spore immobilization and microelectrode positioning, spores were allowed to settle for 30 minutes to ensure that ATP flux measurements were not the result of touch or other movement-induced stimuli.

**Figure S2**. Calibration of ATP electrodes in the presence of 1 mM organic acid (ascorbic acid) or 1 mM amino acid (citrate). The average calibration with ATP only (4.73±1.4 pA/nM) was not significantly different than the calibration in the presence of ascorbic acid (4.63±2.0 pA/nM) or citrate (4.38±2.1 pA/nM). Error bars represent standard deviation of the arithmetic mean (n=3).

**Figure S3.** Calibration of ATP electrodes prior to experimental measurement of spore physiology and after experiments. The average calibration prior to experiments (4.73±1.4 pA/nM) was not significantly different than the calibration after experiments (4.53±2.2 pA/nM). Error bars represent standard deviation of the arithmetic mean for electrodes in four different days of experimentation (n=4).

**Figure S4.** Calibration in the presence of BFA (top) and GdCl_3_ (bottom) show a slight baseline shift in oxidative current, but no effect on calibration slope. All data was corrected for this baseline shift. Error bars represent standard deviation of the arithmetic mean (n=3).


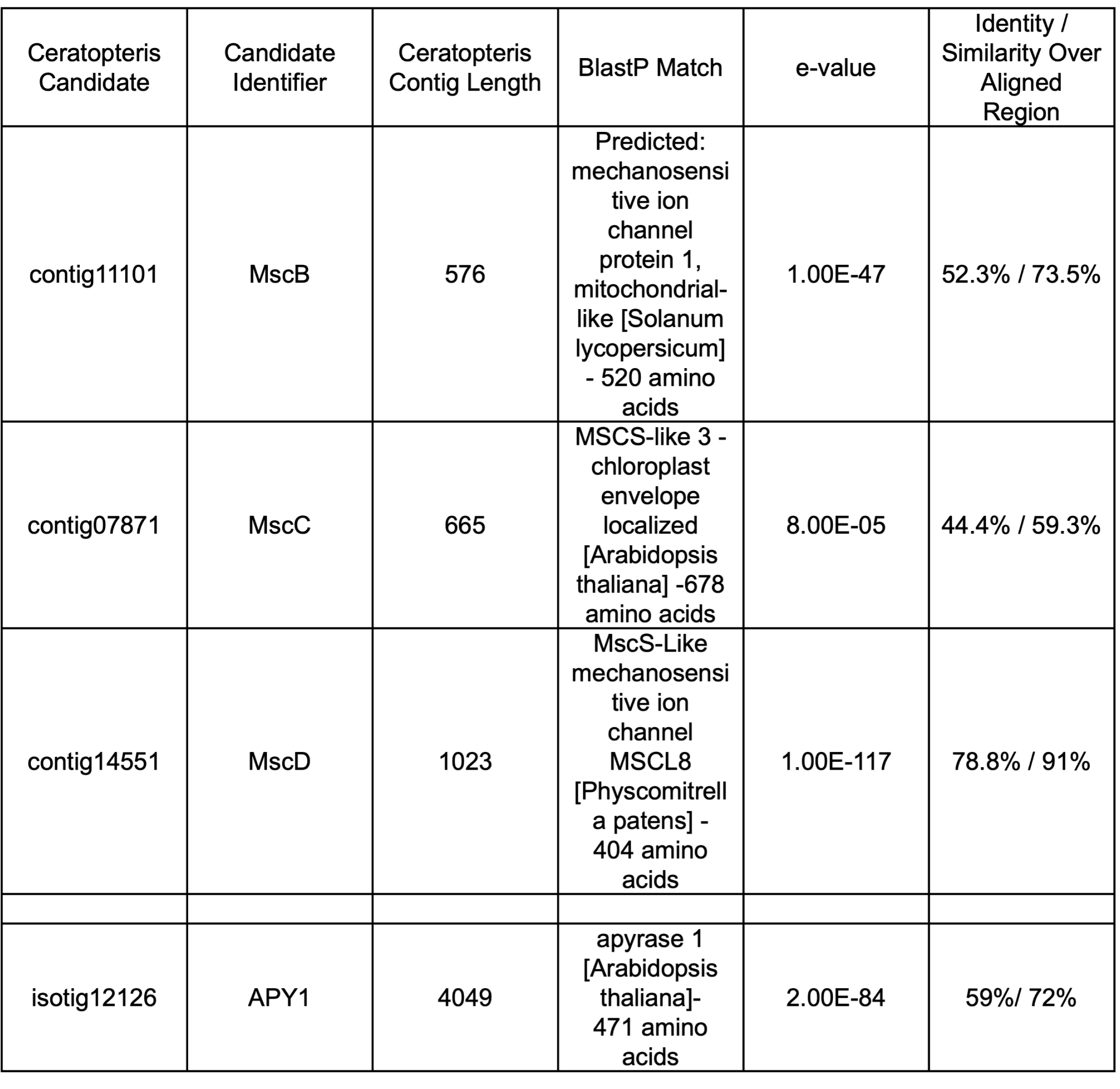


**Table S1:** A 454 analysis of transcripts present after 10 h of light exposure identified sequences that aligned well with apyrase enzymes and MS channels.

|  |
| --- |
| cov pid  **1** **[ . . . . : . . .** **80**  1 C.richardii 100.0% 100.0% **MCILQLHQVGCS-DVYKEFNPAQGQWL----LLKPRCHIHLRASHSRLDCSLNNKRSSWFGISKHSGGSHIPIIHGRKPL**  2 Arabidopsis 80.1% 37.5% **------------------------------------MRRRLHISNGPLSLGVPLGQHGF--------SNILLSNYLRRPI**  3 Chlamydomonas 54.6% 16.4% **MI------MSSTINL-------SGRR-QATSVRQP--------GHSALSCAAMP---RFVGER--LRSREAPTAARRRTV**  4 Oryza 82.5% 34.2% **------MAVGVTSQLFQGVTA-TNRFCQTNKFRNPDIRSSLT--S-TSLSSVPNGHNCW--------GHNILER-NYRPM**  consensus/100% **............................................p.....uh.....ta........up.h.....h+sh**  consensus/90% **............................................p.....uh.....ta........up.h.....h+sh**  consensus/80% **............................................p.....uh.....ta........up.h.....h+sh**  consensus/70% **........huso.pl.......psph.....hhpP.h+.pLp.upusLssulsstppsa........uppl.pthtRRPl**  cov pid  **81**  **. 1 . . . . : .** **160**  1 C.richardii 100.0% 100.0% **LCAASSLISFPSKGFA--------TTAKDLARSSLA--------------------SILKIVQESELFRQCAIPATVIVF**  2 Arabidopsis 80.1% 37.5% **CSVPCRTTAFRCHSFS--------ASGK-AIEPAVKAVTVVL---T----------KSHGLMQQFPFVYKLVPAVALLVF**  3 Chlamydomonas 54.6% 16.4% **TCAAHKGAAAAAVAATATATVSAPPPGPPGRAPPTQDPSVILHLLTSPFISMLGGESSWQYYLFSRVTHEVVAVALLAVF**  4 Oryza 82.5% 34.2% **LYVPSRYRALGVRSFA--------LPVSLQEIPLVKSTSVAL---T----------RSCDTLLANPATALVVPAIGIIVF**  consensus/100% **h.sspphhuh.shuhs.........ss.....s.ht....................p.hthh....hhh.hs.shhlhVF**  consensus/90% **h.sspphhuh.shuhs.........ss.....s.ht....................p.hthh....hhh.hs.shhlhVF**  consensus/80% **h.sspphhuh.shuhs.........ss.....s.ht....................p.hthh....hhh.hs.shhlhVF**  consensus/70% **hssss+htAhss+uFu........ssup.tttPslpssoVhL...T..........pShphh.ts.hstplVssssllVF**  cov pid **161**  **. . . 2 . . . .** **240**  1 C.richardii 100.0% 100.0% **TLWGLGPLLRVIHRASRQGDDTSWK-DSQMYYIFR----SFVRPVLLWVGVIFVCRA-------------FDPLVLST--**  2 Arabidopsis 80.1% 37.5% **SLWGLVPFARQGRNILLNKNDNGWK-KSGTYHVMT----SYVQPLLLWLGALFICRA-------------LDPVVLPT--**  3 Chlamydomonas 54.6% 16.4% **AYWLLTKMAHGSAQAMEKHRDTAGSAGGGEFLRLAADALTGAQAKLFFSVVLFVNVARNSLYIMDGFITKFNPKLPNDWL**  4 Oryza 82.5% 34.2% **ALWGFLPLMRDIRNRF--DHGGNWK-KSPTYLIST----SYLQPLLLWTGATLICRA-------------LDPVVLPS--**  consensus/100% **shWhhh.hh+..tph...tpssshp.tu..ahh.t....ohhpshLha.sshhlshA.............hsPhl.ss..**  consensus/90% **shWhhh.hh+..tph...tpssshp.tu..ahh.t....ohhpshLha.sshhlshA.............hsPhl.ss..**  consensus/80% **shWhhh.hh+..tph...tpssshp.tu..ahh.t....ohhpshLha.sshhlshA.............hsPhl.ss..**  consensus/70% **uLWGLsPhhRsh+pth.pccDsuWK.cSspYhlhs....SalQPlLLWsGslFlCRA.............hDPlVLso..**  cov pid **241**  **: . . . . 3 . .** **320**  1 C.richardii 100.0% 100.0% **--------ETSQAIKQRFLNFVRSLATVLT-----------FAQCSISISHQMQKGSSDGQSSQESRSLGAQFVNNTVYT**  2 Arabidopsis 80.1% 37.5% **--------EASKIVKDRLLNFVRSLSTVLA-----------FAYCLSSLIQQTQKLFSETSNPSDTRNMGFQFAGKALYS**  3 Chlamydomonas 54.6% 16.4% **DDLIRLAVDCLAPLDNVLMKLSLIGTALFGCAVCLRWKDVLVAYGVKTYLEQLDKGQE----------LVQNFINPASNL**  4 Oryza 82.5% 34.2% **--------AASQAVKTRLVTFVRSLSTVLA-----------IAYILTSLIQQLQKFLMDMRNPNDSRRMGFDFAVKAVYT**  consensus/100% **........ts.t.lcphhhphsh.hsslhs...........hA.h..oh.pQhpKh............hs.pFhs.s...**  consensus/90% **........ts.t.lcphhhphsh.hsslhs...........hA.h..oh.pQhpKh............hs.pFhs.s...**  consensus/80% **........ts.t.lcphhhphsh.hsslhs...........hA.h..oh.pQhpKh............hs.pFhs.s...**  consensus/70% **........-sSpslKsRLlsFVRSLoTVLu...........hAYslpSllpQhQKh.p-hpssp-oRphGhpFsspAlYo**  cov pid **321**  **. . : . . . . 4** **400**  1 C.richardii 100.0% 100.0% **AVWVAA---GCLFMELLGFSTQRWLTAGGLGTVLITLAGREIFTNFLSSIMIHATRPFVLNEWIQTKIDG----YEVSGT**  2 Arabidopsis 80.1% 37.5% **AVWVAA---VSLFMELLGFSTQKWLTAGGLGTVLITLAGREILTNFLSSVMIHATRPFVLNEWIQTKIEG----YEVSGT**  3 Chlamydomonas 54.6% 16.4% **LNWVIIVLSALWLAVALGFNLKPLLAVGGASGIIIGLATQQVLGNFVSGLNIFLSRPFVAGEFISLVSQTLSSQTNISGR**  4 Oryza 82.5% 34.2% **GIWIAA---ISLFMELLGFNTQKWITAGGFGTVLLTLAGREIFTNFLSSVMINATRPFVVNEWINTKIDG----VEVSGI**  consensus/100% **h.Wlhh...h.hhh.hLGFshp.hlssGGhuslllsLAspplhsNFlSul.I.hoRPFVhsEaIphh.ps....hplSGh**  consensus/90% **h.Wlhh...h.hhh.hLGFshp.hlssGGhuslllsLAspplhsNFlSul.I.hoRPFVhsEaIphh.ps....hplSGh**  consensus/80% **h.Wlhh...h.hhh.hLGFshp.hlssGGhuslllsLAspplhsNFlSul.I.hoRPFVhsEaIphh.ps....hplSGh**  consensus/70% **ulWVAA...ssLFMELLGFsTQ+WLTAGGhGTVLITLAGREIhTNFLSSlMIaATRPFVlNEWIpTKI-G....hEVSGp**  cov pid **401**  **. . . . : . . .** **480**  1 C.richardii 100.0% 100.0% **VEHVGWWSPTIIRGDDREAVHIPNHKFTVSIVRNLSQKSHWRIKTHFGIS-------------HLDVSKVPSIVADMRKV**  2 Arabidopsis 80.1% 37.5% **VEHVGWWSPTIIRGEDREAIHIPNHKFTVNVVRNLTQKTHWRIKTHLAIS-------------HLDVNKINNIVADMRKV**  3 Chlamydomonas 54.6% 16.4% **VIRIDP-MRTLIATEDGATVTVPNQIIAVSIVVNRSRSPHWTVSTASPLLANTRELRWRMKLPHAALERIEELEAKIDAA**  4 Oryza 82.5% 34.2% **VEHVGWWSPTIIRGDDREAIYIPNHKFTVSILRNNTQRTHWRIKTYLALS-------------HMDAAKIGIIVADMRKV**  consensus/100% **V.+ls....TlIts-DttslhlPNphhsVsllhN.oppsHWplpTh.sl..............Hhsht+l..l.Achcts**  consensus/90% **V.+ls....TlIts-DttslhlPNphhsVsllhN.oppsHWplpTh.sl..............Hhsht+l..l.Achcts**  consensus/80% **V.+ls....TlIts-DttslhlPNphhsVsllhN.oppsHWplpTh.sl..............Hhsht+l..l.Achcts**  consensus/70% **VEHVGWWSPTIIRG-DREAlaIPNHKFTVSIVRNhoQ+oHWRIKTahulS.............HhDlsKIspIVADMRKV**  cov pid **481**  **. 5 . . . . : .** **560**  1 C.richardii 100.0% 100.0% **LAK-HPQVEQR----RLHRRVFFDNINPENQAIMVLVSCFVKTPHFEEYLRVKEVILLDLLKVISHHNARLATPIRSVQR**  2 Arabidopsis 80.1% 37.5% **LAK-NPMVEQQ----RLHRRVFLENVIPENQALSILISCFVKTSHHEEYLGVKEAILLDLLRVISHHRARLATPIRTIRK**  3 Chlamydomonas 54.6% 16.4% **LNAALPQAQVRYSPPDLHLVKFNEG-GAEIAAKVNLVWRLREGGATKELVEK-----------QQTQEMLVQTALLALQK**  4 Oryza 82.5% 34.2% **LAK-NPHIEQQ----RLHRRVFFEKIDPKTQALMIYISCFVKTSHFEEYLNVQEAVMLDLLRIVGHHRARLATQIRTVQK**  consensus/100% **Lst..P.hp.p....cLHhhhF.-t..sc.tAh..hl.phhcssthcEhlth............tppphhltT.lhslp+**  consensus/90% **Lst..P.hp.p....cLHhhhF.-t..sc.tAh..hl.phhcssthcEhlth............tppphhltT.lhslp+**  consensus/80% **Lst..P.hp.p....cLHhhhF.-t..sc.tAh..hl.phhcssthcEhlth............tppphhltT.lhslp+**  consensus/70% **LAK.pPplEQp....RLHRRVFhEslsPEsQAlhlLlSCFVKTuHaEEYLpVpEslhLDLL+lluHHcARLATsIRolQK**  cov pid **561**  **. . . 6 . . . .** **640**  1 C.richardii 100.0% 100.0% **VLDDTASAYRDMNR--PAEAQGRPYLL-VEATAVSNGQRDSEQSGTLKNGASNAKEGGGRTEVSDSSSGNENLESLTDSR**  2 Arabidopsis 80.1% 37.5% **MYTETDVENTPFGESMYGGVTSRRPLMLIEPAYKINGEDKSKSQNRAAKPTAEQ-ENKGSNPKS----------------**  3 Chlamydomonas 54.6% 16.4% **VVRSCDGAFITV--------------------------------------------------------------------**  4 Oryza 82.5% 34.2% **SYGNADIDNIPFGEEMYSRVRGRPLLI--DTSARISDD-KSKP-----RPASRE-DHKVKTVTS----------------**  consensus/100% **.htpss.t.hsh....................................................................**  consensus/90% **.htpss.t.hsh....................................................................**  consensus/80% **.htpss.t.hsh....................................................................**  consensus/70% **shsssDss.hshsc...utspuR..Lh..-sshh.ssp.cSc......pssupt.-ttsps.hS................**  cov pid **641**  **: . . . . 7 . .** **720**  1 C.richardii 100.0% 100.0% **VPSKHKPQLEGLDSMGLNSKDITLLGAAFEKPPAHIPESSEDHNQLYDHTAVKPVSESSPYKHNKVSIKTPEQPVESRVA**  2 Arabidopsis 80.1% 37.5% **------------------------------------------------------KETSSPDLKANVKVGESP--------**  3 Chlamydomonas 54.6% 16.4% **--------------------------------------------------------------------------------**  4 Oryza 82.5% 34.2% **------------------------------------------------------AEAKSASA-DNASISNSE--------**  consensus/100% **................................................................................**  consensus/90% **................................................................................**  consensus/80% **................................................................................**  consensus/70% **......................................................hptpSs.h.spspltps.........**  cov pid **721**  **. . : . . . . 8** **800**  1 C.richardii 100.0% 100.0% **DQEKNHIRPTEGSLQQTGKNYQRPVSNGSSHLDSMQSGEGDCAQQEELKEPWKEEAEARLDTKKSESAVPRGSQSKLDIG**  2 Arabidopsis 80.1% 37.5% **-VSDTNKVPEETVAKP---------------------VIK---AVSKPPTPKDTE----TSGTEKPKAKRSGGTIKSTK-**  3 Chlamydomonas 54.6% 16.4% **--------------------------------------------------------------------------------**  4 Oryza 82.5% 34.2% **KQEQKKSVPEDGRMKN---------------------SKNDHATTTSPSSPWSENMDPIASTSKTGKGKTQGAEATEREG**  consensus/100% **................................................................................**  consensus/90% **................................................................................**  consensus/80% **................................................................................**  consensus/70% **..pppp.hPp-s.hp......................s.t...t.pp..pPhppp....hsspcp.puh.pGup.p.p..**  cov pid **801**  **. . . . : . . .** **880**  1 C.richardii 100.0% 100.0% **--RGSGAVNSSATKSQLEENLVLGVALDGPKRTLPLDEVAPVAQQKE---LVALHNSNSSTTKERRDASPTASQNASDSK**  2 Arabidopsis 80.1% 37.5% **-TDETDSSTSSASRSTLEENIVLGVALEGSKRTLPIEEEIHSPPMETDAKE--LTGARR-SG---GNGPLVADKEQKDSQ**  3 Chlamydomonas 54.6% 16.4% **--------------------------------------------------------------------------------**  4 Oryza 82.5% 34.2% **DGAVSVANSKKESRPVFEDNIVLGLALEGSKRTLPIDDGMNPHLSLSETEQDTVEAASSPKD---KKGQEKGDQRNLDR-**  consensus/100% **................................................................................**  consensus/90% **................................................................................**  consensus/80% **................................................................................**  consensus/70% **..t.osussppto+s.hE-NlVLGlAL-GsKRTLPl--.h.s....p......lpsupp.ps...tpu..husppt.Dp.**    cov pid **881**  **.]** **891**  1 C.richardii 100.0% 100.0% **EQ*--------**  2 Arabidopsis 80.1% 37.5% **SQPNSGASTEP**  3 Chlamydomonas 54.6% 16.4% **-----------**  4 Oryza 82.5% 34.2% **-----------**  consensus/100% **...........**  consensus/90% **...........**  consensus/80% **...........**  consensus/70% **...........** |
|  |

**Figure S5:** Basic alignment between *Ceratopteris* MscB and the most similar sequence in *Arabidopsis thaliana*, *Chlamydomonas reinhardtii*, and *Oryza sativa*. The identities are normalized by alignment length and colored by identity. The alignment was generated using CLUSTAL W (Larkin et al., 2007) and the view was generated using MView (Brown et al., 1998). (cov = coverage and pdi = percent identity/similarity when compared to Ceratopteris)

|  |
| --- |
| cov pid  **1** **[ . . . . : . . .** **80**  1 C.richardii 100.0% 100.0% **-MMKLSRLRNITYYVRDSRECFRFLCGSPSS-------SFSTSN-------FQKSDSIARSSPSVSLPRT-RLLFPGSSN**  2 Arabidopsis_thaliana_pdb\|6VXM\|A 78.7% 40.5% **--------------------------------------------------------------------------------**  3 Chlamydomonas 82.4% 16.8% **------------------------------------------------------------------MIMSSTINLSGRRQ**  4 Oryza 94.8% 34.3% **MSMIAATLRRSS-RVTGSQN-IMEICLGPCVSSGASSRWFSSCTKHSNTSILNQIKAVDRYSPVNGMSMISRVPLSAHMD**  consensus/100% **................................................................................**  consensus/90% **................................................................................**  consensus/80% **................................................................................**  consensus/70% **..................................................................h.h..pl.hsup.p**  cov pid  **81**  **. 1 . . . . : .** **160**  1 C.richardii 100.0% 100.0% **-AQQLLRKPRFHD--FSLIPCYYVHTRSF------------FFNS-KGNTAPEAS-----TTIPSPPSPPSVDPPKEDAQ**  2 Arabidopsis_thaliana_pdb\|6VXM\|A 78.7% 40.5% **------------------------------------------MSS--------KSDDFG-SIVASGV-TGSGDGNGNGND**  3 Chlamydomonas 82.4% 16.8% **--ATSVRQPGHSALSCAAMPRFVGERLRSREAPTAARRRTVTCAAHKGAAAAAVAATATATVSAPPPGPPGRAPPTQDPS**  4 Oryza 94.8% 34.3% **TNWLSTSNPRFNA-----LPGFLGASSICR-----------AYSSDTGIKAEVPQNTVS-N-VPSTE-TVALGTSDGGSS**  consensus/100% **..........................................hsu.........t.....s..sss..ssuhsssttssp**  consensus/90% **..........................................hsu.........t.....s..sss..ssuhsssttssp**  consensus/80% **..........................................hsu.........t.....s..sss..ssuhsssttssp**  consensus/70% **.....hppPtaps.....hPtahstp...............hhuS.pG.tA..susshs.ohlsSss.ssuhsssspsss**  cov pid **161**  **. . . 2 . . . .** **240**  1 C.richardii 100.0% 100.0% **-------------------FL-------TKELVDAANNAAISLRETFISSTTWIH-----ETVEQVLRVLR--DALGYPE**  2 Arabidopsis_thaliana_pdb\|6VXM\|A 78.7% 40.5% **-------------------WVEKA-KDVLQTSVDAVTETAKKT-----------K-----DVSDEMIPHVQ--QFLDSNP**  3 Chlamydomonas 82.4% 16.8% **VILHLLTSPFISMLGGESSWQYYLFSRVTHEVVAVAL---------LAVFAYWLLTKMAHGSAQAMEKHRDTAGSAGGGE**  4 Oryza 94.8% 34.3% **-------------------WIDIF-DNARKCTLDATTDAGKKV-----------K-----ELTDAITPHVQ--QFFDANP**  consensus/100% **...................a........hpp.lsss..................h.....t.spth..hhp..t.hs.s.**  consensus/90% **...................a........hpp.lsss..................h.....t.spth..hhp..t.hs.s.**  consensus/80% **...................a........hpp.lsss..................h.....t.spth..hhp..t.hs.s.**  consensus/70% **...................Wl.hh.ppsp+psVDAsspsuhph...........+.....-ss-thh.Hlp..phhsus.**    cov pid **241**  **: . . . . 3 . .** **320**  1 C.richardii 100.0% 100.0% **YTTETATQFLYTTIAALLVWLVMPRVFRMLHRYFEDGSSLIL---RRSERIPYEVSFWSALENPTKVFVAVVAFSQLGSL**  2 Arabidopsis_thaliana_pdb\|6VXM\|A 78.7% 40.5% **YLKDVIVPVSLTMTGTLFAWVVMPRILRRFHTYAMQSSAKLLPVGFSNEDVPYEKSFWGALEDPARYLVTFIAFAQIAAM**  3 Chlamydomonas 82.4% 16.8% **FLRLAADALTGAQ-AKLFFSVVLFVNVARNSLYIMDGFITKF--------NPKLPNDW--LDDLIRLAVDCL--APLDNV**  4 Oryza 94.8% 34.3% **NLEKVVVPLGGTIFGTMMAWFVMPIVLRRIHKYSIQSPISALLGSSTKNDVSYETSLWSALEDPAKYLITFMAFSEMAGF**  consensus/100% **.hp.shs.h.hs..uthhh.hVh.h.hth.phY..pu...hh.........sh..s.W..L-s.h+hhlshh..u.hssh**  consensus/90% **.hp.shs.h.hs..uthhh.hVh.h.hth.phY..pu...hh.........sh..s.W..L-s.h+hhlshh..u.hssh**  consensus/80% **.hp.shs.h.hs..uthhh.hVh.h.hth.phY..pu...hh.........sh..s.W..L-s.h+hhlshh..u.hssh**  consensus/70% **aLccsssslshThhusLhsWlVMPhllRRhHpYhhpushphL....pppclPYEsShWuALEDPs+hhVshlAFupluuh**  cov pid **321**  **. . : . . . . 4** **400**  1 C.richardii 100.0% 100.0% **VAPTTIASQYLYQIWKGSAVISLVWFLHQWKSSVFRRVLA-----AQNLGSTEREFFHTVEKISSIGLLILGGMGVAETC**  2 Arabidopsis_thaliana_pdb\|6VXM\|A 78.7% 40.5% **VAPTTIAAQYFSPTVKGAVILSLVWFLYRWKTNVITRMLS-----AKSFGGLDREKVLTLDKVSSVGLFAIGLMASAEAC**  3 Chlamydomonas 82.4% 16.8% **---------LMKLSLIGTALFG-CAVCLRWKDVLVAYGVKTYLEQLDKGQELVQNFINPASNLLNWVIIVLSALWLAVAL**  4 Oryza 94.8% 34.3% **TAPSI--SAYLPQAWRGAIVLSFVWFLHRWKTNFITKVAA---------SSIDQTRLSAFDKISSLGLIALGVMALAEAC**  consensus/100% **.........hh...hhGshlhu.shhhhpWKsshhthhht.........tth.pphh.shppl.shslhhluhhh.A.sh**  consensus/90% **.........hh...hhGshlhu.shhhhpWKsshhthhht.........tth.pphh.shppl.shslhhluhhh.A.sh**  consensus/80% **.........hh...hhGshlhu.shhhhpWKsshhthhht.........tth.pphh.shppl.shslhhluhhh.A.sh**  consensus/70% **sAPoh..utYh..sh+GusllShVWFLaRWKoslls+slu.....hpphuul-pphlpsh-KlSSlGLlsLGsMulAEAC**  cov pid **401**  **. . . . : . . .** **480**  1 C.richardii 100.0% 100.0% **GVAVQSILTVGGLGGVATAFAARDILGNMLSGFALQLMKPFSIGDTIKA--------GNVEGQVVDIGVTSTRLLDLDKF**  2 Arabidopsis_thaliana_pdb\|6VXM\|A 78.7% 40.5% **GVAVQSILTVGGVGGVATAFAARDILGNVLSGLSMQFSRPFSMGDTIKA--------GSVEGQVIEMGLTTTSLLNAEKF**  3 Chlamydomonas 82.4% 16.8% **GFNLKPLLAVGGASGIIIGLATQQVLGNFVSGLNIFLSRPFVAGEFISLVSQTLSSQTNISGRVIRIDPMRTLIATEDGA**  4 Oryza 94.8% 34.3% **GVAAQSILTVGGVGGVATAFAARDVLGNMLSGFSLQFSSPFKAGEYIKA--------GSIEGKVIEIGLTSTELMNPEQL**  consensus/100% **GhshpslLsVGGhuGlhhuhAspplLGNhlSGhsh.h.pPF.hG-hIph........sslpGpVlchs.hpT.lhs.-th**  consensus/90% **GhshpslLsVGGhuGlhhuhAspplLGNhlSGhsh.h.pPF.hG-hIph........sslpGpVlchs.hpT.lhs.-th**  consensus/80% **GhshpslLsVGGhuGlhhuhAspplLGNhlSGhsh.h.pPF.hG-hIph........sslpGpVlchs.hpT.lhs.-th**  consensus/70% **GVAlQSILTVGGlGGVATAFAARDlLGNhLSGhulQhS+PFshG-hIKA........GslEGpVI-IGlToTpLhs.-ph**    cov pid **481**  **. 5 . . . . : .** **560**  1 C.richardii 100.0% 100.0% **PVTVPNSFFSSQAIVNKSRATWRSFTLKIPIQLT-------------DFEKVPQITEEVKNMLKSH-PNVTFQNG--VPL**  2 Arabidopsis_thaliana_pdb\|6VXM\|A 78.7% 40.5% **PVLVPNSLFSSQVIVNKSRAQWRAIASKIPLQID-------------DLDMIPQISNEIKEMLRSN-TKVFLGKE--APH**  3 Chlamydomonas 82.4% 16.8% **TVTVPNQIIAVSIVVNRSRSPHWTVSTASPLLANTRELRWRMKLPHAALERIEELEAKIDAALNAALPQAQV--RYSPPD**  4 Oryza 94.8% 34.3% **PVTVPNSLFSSQVIVNRSRAKWRSNVTKIPIRIE-------------DIEKVPAISEEIKVMLRSN-PKVVLDSEAPAPY**  consensus/100% **sVhVPNphhusphlVN+SRu.ahs.s.t.Pl.hp.............sh-hl.tlptclc.hLput.sps.h..t..sP.**  consensus/90% **sVhVPNphhusphlVN+SRu.ahs.s.t.Pl.hp.............sh-hl.tlptclc.hLput.sps.h..t..sP.**  consensus/80% **sVhVPNphhusphlVN+SRu.ahs.s.t.Pl.hp.............sh-hl.tlptclc.hLput.sps.h..t..sP.**  consensus/70% **PVTVPNSlFSSQlIVN+SRApWRohsoKIPlpls.............DlE+lPpIopEIKsML+Ss.PpVhltpc..sPh**    cov pid **561**  **. . . 6 . . . ]** **640**  1 C.richardii 100.0% 100.0% **CHASRISGSVLEISVLCNLTFKGRED-----LLSSQ--QDITLQTL-----KIISN-TGAILNAFPPGTLP*--------**  2 Arabidopsis_thaliana_pdb\|6VXM\|A 78.7% 40.5% **CYLSRVEKSFAELTIGCNLIRMGKEE-----LYNTQ--QEVLLEAV-----KIIKK-HGVSLGTTWDNSTLSNSLEVLFQ**  3 Chlamydomonas 82.4% 16.8% **LHLVKFNEGGAEIAAKVNLVWRLREGGATKELVEKQQTQEMLVQTALLALQKVVRSCDGAFITV----------------**  4 Oryza 94.8% 34.3% **CYLSRLESSYGELTIGCNLTKMTKDE-----WLSTT--QGILLEAA-----KIIKL-HGVELGSTTQCC-----------**  consensus/100% **hahs+hptuhhElshhsNLhhhh+-t.....hhppp..Qthhlpsh.....Kllp..pGs.lss................**  consensus/90% **hahs+hptuhhElshhsNLhhhh+-t.....hhppp..Qthhlpsh.....Kllp..pGs.lss................**  consensus/80% **hahs+hptuhhElshhsNLhhhh+-t.....hhppp..Qthhlpsh.....Kllp..pGs.lss................**  consensus/70% **CaLSRlppShuEloltCNLshhs+E-.....LlsoQ..Q-lLLpss.....KII+p.cGs.Lssh..ss...........** |

**Figure S6:** Basic alignment between *Ceratopteris* MscC and the most similar sequence in *Arabidopsis thaliana*, *Chlamydomonas reinhardtii*, and *Oryza sativa*. The identities are normalized by alignment length and colored by identity. The alignment was generated using CLUSTAL W (Larkin et al., 2007) and the view was generated using MView (Brown et al., 1998). (cov = coverage and pdi = percent identity/similarity when compared to Ceratopteris)

|  |
| --- |
| cov pid  **1** **[ . . . . : . . .** **80**  1 C.richardii 100.0% 100.0% **--------------------------------------------------------------------------------**  2 Arabidopsis_thaliana 77.3% 47.7% **--------------------------------------------------------------------------------**  3 Chlamydomonas 43.8% 10.4% **----------------------------------------------------------------MIMSSTINLSGRRQAT**  4 Oryza 40.5% 9.5% **MSMIAATLRRSSRVTGSQNIMEICLGPCVSSGASSRWFSSCTKHSNTSILNQIKAVDRYSPVNGMSMISRVPLSAHMDTN**  consensus/100% **................................................................................**  consensus/90% **................................................................................**  consensus/80% **................................................................................**  consensus/70% **................................................................................**  cov pid  **81**  **. 1 . . . . : .** **160**  1 C.richardii 100.0% 100.0% **--------------------------------------------------------------------------------**  2 Arabidopsis_thaliana 77.3% 47.7% **--------------------------------------------------------------------------------**  3 Chlamydomonas 43.8% 10.4% **SVRQPGHSALSCAAMPRFVGERLRSREAPTAARRRTVTCAAHKGAAAAAVAATATATVSAPPPGPPGRAPPTQDPSVILH**  4 Oryza 40.5% 9.5% **W---LSTSNPRFNALPGFLGASSICRAYSSD-----------TGIKA------------EVPQNTVSNVPSTE-------**  consensus/100% **................................................................................**  consensus/90% **................................................................................**  consensus/80% **................................................................................**  consensus/70% **................................................................................**  cov pid **161**  **. . . 2 . . . .** **240**  1 C.richardii 100.0% 100.0% **--------------------------------------------------------------------------------**  2 Arabidopsis_thaliana 77.3% 47.7% **--------------------------------------------------------------------------------**  3 Chlamydomonas 43.8% 10.4% **LLTSPFISMLGGESSWQYYLFSRVTH---------------------------------EVV----AVALLAVFAYWLLT**  4 Oryza 40.5% 9.5% **---TVALGTSDGGSSWIDIFDNARKCTLDATTDAGKKVKELTDAITPHVQQFFDANPNLEKVVVPLGGTIFGTMMAWFVM**  consensus/100% **................................................................................**  consensus/90% **................................................................................**  consensus/80% **................................................................................**  consensus/70% **................................................................................**  cov pid **241**  **: . . . . 3 . .** **320**  1 C.richardii 100.0% 100.0% **--------------------------------------------------------------------------------**  2 Arabidopsis_thaliana 77.3% 47.7% **--------------------------------------------------------------------------------**  3 Chlamydomonas 43.8% 10.4% **KMAHGSAQAMEKHRDTAGSAGGGEFLRLAADALTGAQ--AKLFFSVVLFVNVARNSLY--------IMDGFIT-KFNPKL**  4 Oryza 40.5% 9.5% **PIVLRRIHKYSIQSP--------------ISALLGSSTKNDVSYETSLWSALEDPAKYLITFMAFSEMAGFTAPSISAYL**  consensus/100% **................................................................................**  consensus/90% **................................................................................**  consensus/80% **................................................................................**  consensus/70% **................................................................................**  cov pid **321**  **. . : . . . . 4** **400**  1 C.richardii 100.0% 100.0% **---------MTFARCSMSL-------------------SHQMQKVISGQGS-------QESRSLGAQFVNNTLYTAVWV-**  2 Arabidopsis_thaliana 77.3% 47.7% **---------------------------------------------------------------MGFSFAGKAVYTAAWV-**  3 Chlamydomonas 43.8% 10.4% **PNDWLDDLIRLAVDCLAPLDNVLMKLSLIGTALFGCAVCLRWKDVLVAYGVKTYLEQLDKGQELVQNFINPASNLLNWVI**  4 Oryza 40.5% 9.5% **PQAWRGAIVLSF-----------------------VWFLHRWKTNFITKVAASSI---------DQTRLSAFDKISSLGL**  consensus/100% **................................................................s.phhs.h..h.shs.**  consensus/90% **................................................................s.phhs.h..h.shs.**  consensus/80% **................................................................s.phhs.h..h.shs.**  consensus/70% **.........h.h...........................hphppsh.s.ss............hstsFlsssshhusWV.**  cov pid **401**  **. . . . : . . .** **480**  1 C.richardii 100.0% 100.0% **--AAVCLFMELLGFSTQKWLTAGGLGTVLLTLAGREIFTNFLSSIMIHATRPFVLNEWIQTKID----GYEVSGTVEHVG**  2 Arabidopsis_thaliana 77.3% 47.7% **--AAASLFMELLGFSTQKWLTAGGLGTVLLTLAGREILTNFLSSIMIHATRPFVLNEWIQTKIG----GYEVSGTVEQVG**  3 Chlamydomonas 43.8% 10.4% **IVLSALWLAVALGFNLKPLLAVGGASGIIIGLATQQVLGNFVSGLNIFLSRPFVAGEFISLVSQTLSSQTNISGRVIRID**  4 Oryza 40.5% 9.5% **IALGVMALAEACGVAAQSILTVGGVGGVATAFAARDVLGNMLSGFSLQFSSPFKAGEYIKAG--------SIEGKVIEIG**  consensus/100% **..hus.hhh.hhGhshp.hLssGGhuslhhshAspplhsNhlSuh.l.hopPFhhsEaIphh........plpGpV.pls**  consensus/90% **..hus.hhh.hhGhshp.hLssGGhuslhhshAspplhsNhlSuh.l.hopPFhhsEaIphh........plpGpV.pls**  consensus/80% **..hus.hhh.hhGhshp.hLssGGhuslhhshAspplhsNhlSuh.l.hopPFhhsEaIphh........plpGpV.pls**  consensus/70% **..hushhhhEhLGFusQphLTsGGlGsVllsLAuR-lLsNFLSul.IahoRPFVhsEaIpst.t....thplSGpV.clG**    cov pid **481**  **. 5 . . . . : .** **560**  1 C.richardii 100.0% 100.0% **WWSPTIIRGDDREAVHIPNHKFTVSIVRNLTQKSHWRIKTHFGI-------------SHLDVSKVPNIVADMRKVLAKH-**  2 Arabidopsis_thaliana 77.3% 47.7% **WWSPTIIRGDDREAVHIPNHQFSVNIVRNLTQKTHWRIKTHLAI-------------SHLDVSKINNIVADMRKVLSKN-**  3 Chlamydomonas 43.8% 10.4% **PMR-TLIATEDGATVTVPNQIIAVSIVVNRSRSPHWTVSTASPLLANTRELRWRMKLPHAALERIEELEAKIDAALNAAL**  4 Oryza 40.5% 9.5% **LTS-TELMNPEQLPVTVPNSLFSSQVIVNRSRAKWRSNVTKIPIRIE-------------DIEKVPAISEEIKVMLRSN-**  consensus/100% **.hp.T.lhs.-t.sVplPNp.hsspllhNhopt.ahp..Tt.sl................slp+l.tl.tchchhLttt.**  consensus/90% **.hp.T.lhs.-t.sVplPNp.hsspllhNhopt.ahp..Tt.sl................slp+l.tl.tchchhLttt.**  consensus/80% **.hp.T.lhs.-t.sVplPNp.hsspllhNhopt.ahp..Tt.sl................slp+l.tl.tchchhLttt.**  consensus/70% **hhS.TlIts-DptsVplPNphFoVsIVhNhoppsHWplpT+hsI.............sHhDlpKlssIsA-h+tsLsps.**  cov pid **561**  **. . . 6 . . . .** **640**  1 C.richardii 100.0% 100.0% **PQVEQRRLHRRVFFETIDPENQAIMILVSCFVKTSHFEEYLRVKEVILLDLLK-------VISHHHARLATPIRSVQRVQ**  2 Arabidopsis_thaliana 77.3% 47.7% **PQIEQQKIHRRVFLEDIDPENQALRILISCFVKTSRFEEYLCVKEAVLLDLLT-------VIRHHGARLATPIRTVQRMR**  3 Chlamydomonas 43.8% 10.4% **PQAQVRY--SPPDLHLVKFNEGGAEIA------AKVNLVWRLREGGATKELVEKQQTQEMLVQTALLALQKVVRSCDGA-**  4 Oryza 40.5% 9.5% **PKVVLDSEAPAPYCYLSRLESSYGELTIGCNLTKMTKDEWLSTTQGILLEAAK-------IIKLHGVELGSTTQCC----**  consensus/100% **Pph..p.....s.h...c.ppthh.lh......t.h...ah.hptshhh-hhp.......llphthhtLtpshpss....**  consensus/90% **Pph..p.....s.h...c.ppthh.lh......t.h...ah.hptshhh-hhp.......llphthhtLtpshpss....**  consensus/80% **Pph..p.....s.h...c.ppthh.lh......t.h...ah.hptshhh-hhp.......llphthhtLtpshpss....**  consensus/70% **PQlp.cp.tptsahchlc.EspuhcIhluC.lpspph-EaLpscpulLL-Llc.......lIppHtscLuoslRospth.**  cov pid **641**  **: . . . . 7 . .** **720**  1 C.richardii 100.0% 100.0% **DDVEPRTPPFRDINRSAEALARSYLLVETTAISSGQQ-DGGPSGTSKNGHPDAKEAVAIVEVSEMGPQNEVSESSDESKV**  2 Arabidopsis_thaliana 77.3% 47.7% **NEAEVDTAGFSDIVFNQAAMNRRYMLIEPSYKINSDDNSKSPSPSPGQKSPSPGQKS-----EERDLQEEPSET------**  3 Chlamydomonas 43.8% 10.4% **---------FITV-------------------------------------------------------------------**  4 Oryza 40.5% 9.5% **--------------------------------------------------------------------------------**  consensus/100% **................................................................................**  consensus/90% **................................................................................**  consensus/80% **................................................................................**  consensus/70% **.........F.sl...................................................................**  cov pid **721**  **. . : . . . . 8** **800**  1 C.richardii 100.0% 100.0% **PGKHKTHNEGIVHMGLSSIDISLLGVDFEGPAICMHETLDHSGQSDDKGAVKSD-SEPVQGEDELPSNVHDQSSLSMSFK**  2 Arabidopsis_thaliana 77.3% 47.7% **--KAETENNGSVPVSNAKKE-------NQKA------ALGSNSNTGTKGSSTSTSDQPVAQ-------------------**  3 Chlamydomonas 43.8% 10.4% **--------------------------------------------------------------------------------**  4 Oryza 40.5% 9.5% **--------------------------------------------------------------------------------**  consensus/100% **................................................................................**  consensus/90% **................................................................................**  consensus/80% **................................................................................**  consensus/70% **................................................................................**  cov pid **801**  **. . . . : . . .** **880**  1 C.richardii 100.0% 100.0% **LDKAHTKSGESSSHQLGKKSEKASSKAETNLDSVGLKEEKSSEQQDVQVSPSIQKEEKAGRLNAKQSQFDTMQEPQSELD**  2 Arabidopsis_thaliana 77.3% 47.7% **------KSEEKKKESVGDPH-----KAE--------KDEVSD--DEATIEQTLKSKAKQGS-----------EKNNGESK**  3 Chlamydomonas 43.8% 10.4% **--------------------------------------------------------------------------------**  4 Oryza 40.5% 9.5% **--------------------------------------------------------------------------------**  consensus/100% **................................................................................**  consensus/90% **................................................................................**  consensus/80% **................................................................................**  consensus/70% **................................................................................**  cov pid **881**  **. 9 . . . . : ]** **958**  1 C.richardii 100.0% 100.0% **VDQGLGMNNPPVAKLEENLVLGVALDGPKRTLPLDEVAPSLQHKELVVSRNGNSSTGNERRDRSQVVDSNPTDIRER***  2 Arabidopsis_thaliana 77.3% 47.7% **AR--DGGGSGTSSLLEENLVLGVALDGSKRTLPIDEEHKASGAL--MDS--EELGIGSE-------------------**  3 Chlamydomonas 43.8% 10.4% **------------------------------------------------------------------------------**  4 Oryza 40.5% 9.5% **------------------------------------------------------------------------------**  consensus/100% **..............................................................................**  consensus/90% **..............................................................................**  consensus/80% **..............................................................................**  consensus/70% **..............................................................................** |

**Figure S7:** Basic alignment between *Ceratopteris* MscD and the most similar sequence in *Arabidopsis thaliana*, *Chlamydomonas reinhardtii*, and *Oryza sativa*. The identities are normalized by alignment length and colored by identity. The alignment was generated using CLUSTAL W (Larkin et al., 2007) and the view was generated using MView (Brown et al., 1998). (cov = coverage and pdi = percent identity/similarity when compared to Ceratopteris)

**Figure S8:** Analysis of Msc Candidate Transcript Abundance after 6 hours and 24 hours of light exposure. Target abundance relative to two α-tubulin control genes and normalized to time of initial light exposure (0 hour).
